# Assessing expanded community wide treatment for schistosomiasis: Baseline infection status and self-reported risk factors in three communities from the Greater Accra region, Ghana

**DOI:** 10.1371/journal.pntd.0007973

**Published:** 2020-04-27

**Authors:** Lucas J. Cunningham, Suzy J. Campbell, Samuel Armoo, Artemis Koukounari, Victoria Watson, Pamela Selormey, J. Russell Stothard, Bright Idun, Manfred Asiedu, Yvonne Ashong, Emily R. Adams, Mike Yaw Osei-Atweneboana

**Affiliations:** 1 Department of Tropical Disease Biology, Liverpool School of Tropical Medicine, Liverpool, United Kingdom; 2 Evidence Action, Washington D.C., United States of America; 3 Department of Biomedical and Public Health Research, Council for Scientific and Industrial Research—Water Research Institute, Council Close, Accra, Ghana; 4 Department of Infectious Disease Epidemiology, Faculty of Epidemiology and Population Health, London School of Hygiene & Tropical Medicine, Keppel Street, London, United Kingdom; 5 Department of Biostatistics, University of Liverpool, Liverpool, United Kingdom; 6 Department of Parasitology, Noguchi Memorial Institute for Medical Research, University of Ghana, Legon, Ghana; Ministère de la Santé Publique et de la Lutte contre les Endémies, NIGER

## Abstract

**Background:**

This paper reports on the baseline prevalence and associated risk factor findings of a pilot, longitudinal study exploring community-wide treatment of schistosomiasis and soil-transmitted helminthiasis, using albendazole plus praziquantel in the Greater Accra region of Ghana.

**Method:**

From three communities, at least, 658 individuals were enrolled into the study via random household selection. Prevalence and intensity of schistosomiasis and STH infection were determined from stool and urine samples with a questionnaire being administered in order to explore other morbidities and risk factors. Factor analysis of household demographic variables was undertaken to generate a socioeconomic score; this was then further categorised into tertiles. Proportional-odds cumulative logit generalised estimating equation (GEE) models were used to investigate categorical ordinal intensity of infection associations with morbidity. Separately, logistic GEE models were used to investigate risk factor associations with infection prevalence.

**Results:**

Both *Schistosoma haematobium* and *S*. *mansoni* were prevalent in the three communities, with the prevalence of *S*. *haematobium* ranging from 3.3% (24/679; 95% CI = 1.9–4.7) to 19% (114/632; 95% CI = 15.8–22.2) and *S*. *mansoni* ranging from 30% (202/679; 95% CI = 26.5–33.5) to 78.3% (409/536; 95% CI = 74.7–81.9). The total prevalence of STH across all three sites was negligible at 1.3% (24/1847; 95% CI = 0.8–1.9) comprising mainly hookworm (10/1847). Multivariable statistical models indicated males to be 2.3 (95% CI = 1.7–3.3) times more likely to have a high intensity *S*. *mansoni* infection and 1.5 (95% CI = 1.1–2) times more likely to have a high intensity of *S*. *haematobium* infection compared to females. There was no significant difference in the likelihood of infection with *S*. *mansoni* between adults and school age children (SAC), however *S*. *haematobium* infections were found to be 2.5 (95% CI = 1.8–3.5) times more likely to occur in school age children than in adults.

Multivariable statistical models (adjusted for age and sex) indicated an association between schistosomiasis and a number of self-reported morbidity indicators (notably diarrhoea and blood in stool and urine). Low socio-economic status was also associated with SCH infection (OR: 2; 95% CI = 1.3–3.2).

**Conclusion:**

The communities targeted by this study showed a range of *Schistosoma* prevalence’s of infection, from hypo-endemic through to meso-endemic and hyper-endemic. The prevalence of SCH across the different age groups in the study locations highlights the large number of individuals currently being left out of the standard morbidity control method of annual treatment of the SAC.

## Author summary

In this manuscript we detail the baseline results of a broader study investigating the feasibility of expanding access of albendazole and praziquantel to additional at-risk groups in three communities (Tomefa, Maheam and Torgahkope-Adakope) located around the Weija reservoir in the Greater Accra district of Ghana. Our results show that intestinal schistosomiasis was the most common form of disease across all three sites (44.2%) with Tomefa possessing the highest prevalence at 78.3%. The prevalence of urinary schistosomiasis was found to be 11.9% with the highest prevalence found in Torgahkope-Adakope (19%). STH was found to have a very low prevalence across the three communities with only 1.3% of participants found to be positive. No significant difference in STH prevalence was observed between males (0.92%; 95% CI = 0.4–1.81) and females (1.07%; 95% CI = 0.51–1.95). Statistical analysis revealed that males were more likely to be infected and to have high intensity infections of both forms of schistosomiasis. Pre-school age children (PSAC) were found to be less likely to be infected for either form of schistosomiasis in communities with low to medium prevalence of disease, however in Tomefa, where prevalence of intestinal schistosomiasis is high, there was little difference in the prevalence of disease between PSAC, school-age children (SAC) and adults. Our study shows that there is a need to treat the wider community outside of the current morbidity control strategy of mass-drug administration to SAC.

## Introduction

Schistosomiasis (SCH) and soil transmitted helminthiasis (STH) are two of the 18 listed neglected tropical diseases (NTDs). Combined, they contribute the highest number of DALYs of any NTD and approximately ~90% of all the cases attributed to the 18 NTDs [1]. Globally four main species of STH are responsible for the majority of morbidity (*Ascaris lumbricoides*, *Trichuris trichiura*, *Necator americanus* and *Ancylostoma duodenale*). Five species of *Schistosoma* are responsible for human schistosomiasis, globally, the three most common are *Schistosoma mansoni* (found in 55 countries), *S*. *haematobium* (found in 53 countries) and *S*. *japonicum* (found in China, Indonesia and the Philippines). The global distribution of schistosomiasis is heavily skewed towards sub-Saharan Africa which contributes 93% of all schistosomiasis cases, currently estimated at 192 million cases [2].

The prevalence of STH in sub-Saharan Africa comprise 24–25% of all global infections of the disease, with approximately 300 million cases of *A*. *lumbricoides*, 168 million cases of *T*. *trichiura* and 200 million cases of hookworm [3].

The effects of these parasitic helminths on the host’s body are manifold including fatigue, stunted growth and likely impaired cognitive function [4–6]. This is most acutely observed in children where infection coincides with important developmental stages in the individual where there is both physical and mental development [7]. There is growing evidence that both STH and SCH contribute to higher levels of other infectious diseases such as tuberculosis [8], HIV [9] and giardiasis [10]. The urinary form of schistosomiasis has also been associated with bladder cancer [11]. *Schistosoma haematobium* infection can lead to male or female genital schistosomiasis (MGS and FGS respectively) resulting in menorrhagia, dyspareunia, low fertility and increased risk of contracting sexually transmitted infections [12–14]. Neither form of genital schistosomiasis is well understood but it is believed that FGS is one of the most widespread, undiagnosed, gynaecological conditions in Africa with a conservative estimate predicting that 19 million girls and women will develop FGS by 2023 [15].

The World Health Organization (WHO) recommends that the control efforts focus on reducing morbidity for both SCH and STH by carrying out mass drug administration (MDA) campaigns on school age children (SAC) and if possible on the pre-school age children (PSAC) as well as girls and women of reproductive age [16–19]. The WHO’s push for SCH control has been buoyed by the support of pharmaceutical companies who have pledged their support for NTD control, with Merck having donated over 500 million praziquantel tablets to date, since 2007 [20].

Both SCH and STH are prevalent in Ghana where the main control strategy follows WHO recommendations for reducing childhood morbidity through annual MDA of praziquantel (PZQ) and albendazole; Ghana has been implementing this strategy since 2005 for STH and 2008 for SCH. Whilst indicatively, expanding MDA to include PSAC and adults may improve the effectiveness of SCH and STH control, as left untreated these two non-school cohorts act as a reservoir for disease transmission. Without other interventions such as expanding access to treatment to the whole community, implementation of WASH strategies, and/or snail-control, it will be unlikely to eliminate the transmission of either disease in hyper endemic areas, through MDA alone [21–23]. Although the current evidence available for the impact of such methods on STH is more limited than it is for SCH and this is reflected in the current draft for 2021–2030 roadmap for NTDs [24].

This argument for expanding the access to treatment, to the larger community, has been supported by studies using mathematical models to explore the effectiveness of different treatment strategies targeting STH [25–28]. These studies have suggested that it may be possible to reach the transmission break-point through community wide MDA, however this would need to be at a high coverage (≥75%) [27]. Some of these models have also shown that an annual treatment with high community coverage has the potential to have a similar effect on child morbidity as would biannual treatment of the child cohort. This benefit could also extend to cost, in areas of high-transmission or where school-enrolment is low [28, 29]. Further support for expanding treatment to additional community cohorts comes from the observed evidence that different helminth parasites are found at differing prevalences, and intensities across the various age groups [30]. For example *A*. *lumbricoides* is found in a higher abundance amongst SAC whereas hookworm tends to be harboured, in greater number, by adults [26]. The same is true of the two most important schistosome species, with *S*. *haematobium* found in higher number and intensities in SAC and *S*. *mansoni* affecting both SAC and young adults in equal number and intensity [31–33]. Although it is widely accepted that scale up should be implemented where possible and it is likely going to be required to meet the 2020 goals, to date very few national control programmes have implemented this strategy. This is likely due to the donation of PZQ tablets being too few in number to treat whole communities in at risk areas and the increased complexity of treating a more mobile adult population compared to SAC [34].

Based on the World Health Organisations weekly epidemiological records from 2017 [35] it can be estimated that 57.4 million children in Africa received treatment for schistosomiasis, representing a coverage of 57.2%. A lower number of adults, 9.6 million, were reported to have received treatment, equating to a coverage of 10.9%. The praziquantel coverage of PSAC is not currently included in reports of coverage with regards to control of schistosomiasis. However, it is reported in the coverage of STH treatment with >32 million treatments ascribed to PSAC, resulting in a coverage of 32%. The treatment coverage for STH amongst the SAC population in Africa is estimated to be 66.6% (117.5 million) in 2017. Of the adult population, only women of reproductive age are considered in need of treatment for STH, currently there is no data for Africa and only the global coverage for this cohort has been reported. Of the estimated 688 million women of reproductive age 127 million received treatment, representing a global coverage of 18.5% [35]. It is likely that elimination of SCH and STH as a public health problem would also require a holistic approach. The inclusion of snail control for SCH and the introduction of improved water, sanitation and hygiene (WASH) practices; as health education and behavioural change are key elements to controlling SCH and STH [36].

In Ghana a feasibility and acceptability study is being undertaken, aimed at exploring the scaling up of PZQ and albendazole (ABZ) treatments to include pre-school age children (PSAC), school aged children (SAC) and adults, including pregnant women (APW) [37].

Previous studies had highlighted specific risk factors associated with an increase in the likelihood that an individual would become infected. Notably age and sex are considered major risk factors of schistosomiasis and hookworm infection in both males and individuals >10 years of age [38–40]. Socio economic status has also been implicated with the likelihood of infection, although this was not always found to be the case [39, 40] with better access to clean water, such as piped water, being a major contributor in cases of *S*. *mansoni* [41]. Specific water-contact behaviours have also been identified to have a, significant, positive association with heavy infection, the most important of these behaviours include swimming, bathing and fishing [38, 41–45]. Morbidity indicators have also been identified that correlate with *Schistosoma* infection, although some of these are typically species specific, such as blood in stools and blood in urine being associated with *S*. *mansoni* and *S*. *haematobium* respectively [46–53]. Similar risk factors and morbidity indicators have been identified for STH positive individuals, these include the sex of individuals, lower socio-economic background, reduced access to clean water, stunting, low weight and diarrhoea [40, 54–56].

In this manuscript, the results of the baseline survey are analysed to quantify the prevalence of SCH and STH, to determine the associations between WASH and demographic risk factors with STH and SCH infection, and to investigate the associations between morbidity and the intensities of STH and schistosomiasis infection, in the three study communities, as per the study protocol published previously [37]. In brief this is the first component of the broader study being conducted over the course of at least 18 months with four sampling rounds at baseline, 6 months, 12 months and 18 months, to screen for SCH and STH.

### Primary and secondary aims

The primary aim of this baseline survey is to report the prevalence and intensity of SCH and STH in the three local communities selected.

Additional secondary aims include identifying associations with WASH indicators, demographic risk factors and infection of STH and SCH as well as, self-reported, morbidity indicators associated with heavier intensities of these infections (-whenever this was computationally feasible-).

## Methods

### Ethics

Ethics for the study was obtained from the Liverpool School of Tropical Medicine (research protocol 16–044) and the Council for Scientific and Industrial Research (CSIR), Ghana (RPN 003/CSIR-IRB/2016). All adults enrolled in the study provided informed, written, consent, and written assent for their children.

### Sample sites

Three sites were selected in the Greater Accra region based on national control survey data using Kato-Katz to screen for SCH and STH infections. The sites selected had a minimum SCH prevalence of ≥10% based on school surveys conducted in 2016. The following three communities, Tomefa (longitude: -0.37688, latitude: 5.57309), Manheam (longitude: -0.39127, latitude: 5.55229) and Torgahkope-Adakope (longitude: -0.38176, latitude: 5.6055) were included in the study. All communities are situated around the Weija water reservoir (longitude: -0.360714, latitude: 5.577372). All three sites closely border the reservoir and have low levels of infrastructure with the percentage of households possessing their own latrines making up only 27.3% (95% CI = 25.6–29.1) of households. The percentage of individuals reporting to use a latrine or toilet, either personal or shared, was 57% (95% CI = 55–59), with 42.9% (95% CI = 40.9–44.9) of participants defecating in either the bush, rivers or the reservoir.

### Sample size calculation

The sample size calculation, has been previously described [37] but in brief due to the small community sizes a hypergeometric formula was used assuming an estimated SCH prevalence of 20%.

$${n=Nz}^{2}\rho q/(E^{2}(N-1)+z^{2}\rho q)$$

where: *N* = population size (1500 people); *p* = estimated prevalence (0.2); *q* = 1-*p*; *E* = accuracy of estimation (0.03); *z* = 1.96 (confidence level 95%).

To take into account differences in relevant covariates, such as age and sex, a 40% buffer was applied resulting in a baseline sample size of 658 people per community of 1,500 people.

### Enrolment and random household selection

Each community was sensitised to the project through meetings with village elders. Following this sensitisation process every household in the community was visited and the number of residents enumerated. These data were then used to estimate the number of households needed to recruit the required number of participants based on the sample size calculation.

Households were then randomly selected to be approached for recruitment, using a post-enumeration strategy of selecting odd-numbered houses. Where a household refused to participate or was absent the next enumerated house was selected, in accordance with the protocol [37]. At each household the study was explained, allowing opportunity for questions and provision of written study pamphlets, after which individual written consent was obtained. An individual and household questionnaire was conducted, and two specimen pots provided to each enrolled participant with instructions for collection of faecal and urine specimens. The three communities have estimated population sizes of ~911 for Torgahkope-Adakope, ~1,462 for Tomefa and ~3,954 for Manheam. These figures were calculated based on the average household number for each community multiplied by the total number of enumerated households.

### Parasitological analysis

**Fig 1:**
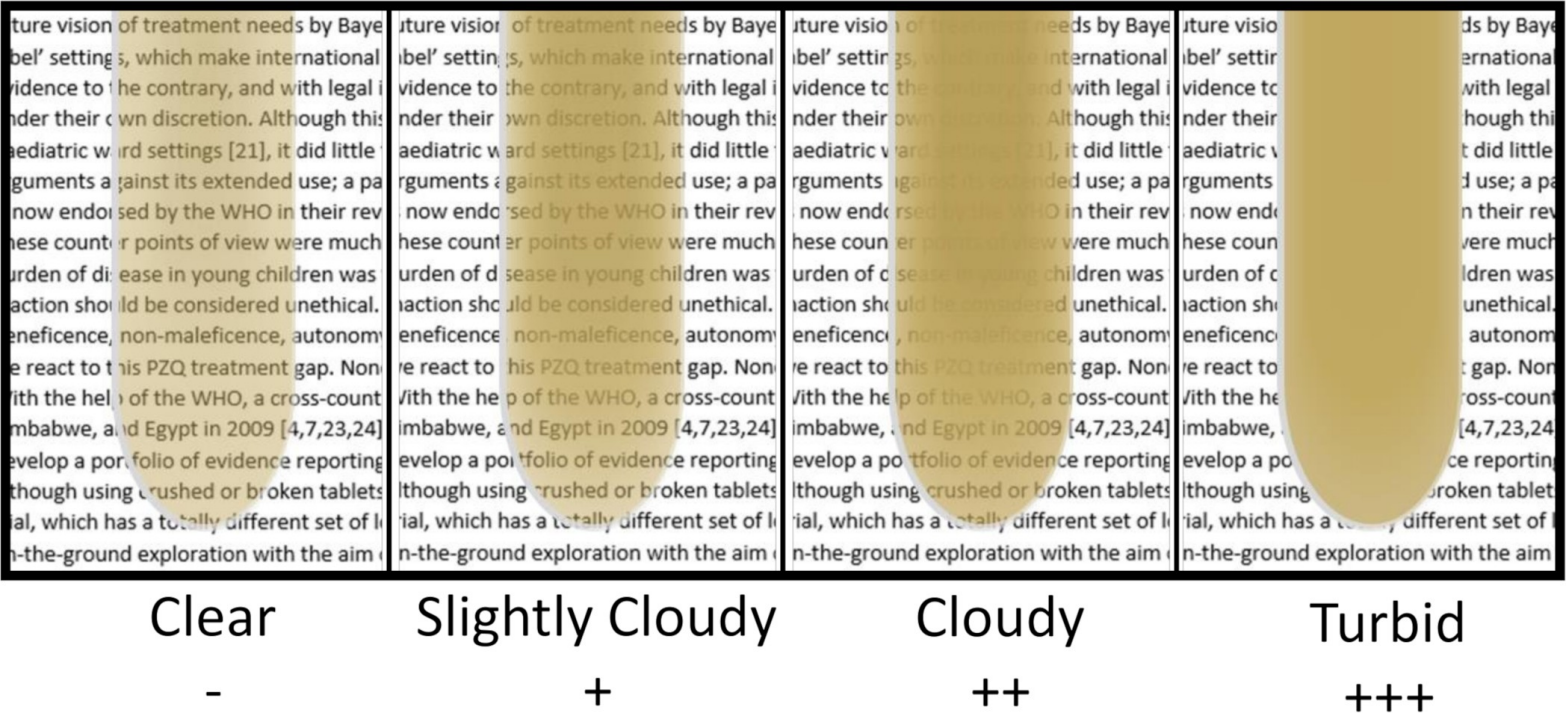
Visual guide used to standardise the assessment of urine turbidity.

The first bowel movement and urination of the day were requested from each enrolled participant for parasitological examination, each participant was requested to supply only a single faecal and urine sample. Faecal and urine samples were examined by trained technicians using two Kato-Katz slides per sample and the filtration of 10mL of urine for each participant. The intensity of infection for the two schistosome species was calculated using the WHO guide (*S*. *mansoni*: light-intensity 1–99 eggs, medium-intensity 100–399 eggs, heavy-intensity > = 400 eggs; *S*. *haematobium*: light-intensity 1–50 eggs, heavy-intensity >50 eggs). The urine samples were also examined visually for haematuria and turbidity and with Hemastix Urine Test Strips for the detection of microhaematuria. The interpretation of the turbid assessment of the urine sample used an intensity scale based on visual guides to standardise the analysis (Fig 1).

A 25 μm pore Plastok (Birkenhead, UK) nylon filter, housed in a Swinnex filter holder, was used to filter 10 mL of urine to screen for both *S*. *haematobium* and *S*. *mansoni* eggs. The Sterlitech (Kent, USA) Kato-Katz kit was used to screen the faecal samples for SCH and STH eggs, two slides were made and examined for each faecal sample. Both the nylon filters and Kato-Katz slides were examined as per WHO recommendations with the inclusion of a 10% quality control check.

### Risk factor analysis

Two sets of questionnaires were administered to each household, the first was answered by the head of the household and covered household-specific characteristics such as household demographics and visual inspection of household water and sanitation facilities S1 Questionnaire). The second questionnaire was administered at an individual level and covered health and hygiene behavioural patterns such as current health symptoms being experienced, history of drug treatment, handwashing practices and participant defecation and urination practices (S2 Questionnaire). For children aged less than 12 years a parent or guardian answered on behalf of the child. All questionnaires used standardised questions used in previous studies [54] and tested in-country with field-staff during training sessions.

### Treatment and exclusion criteria

Treatment was offered to the entire community including those that were not enrolled in the study, after all specimens had been received. The treatment consisted of praziquantel and albendazole tablets that were delivered by trained field staff from The Council for Scientific and Industrial Research (CSIR) and Ghana Health Service Nurses. Praziquantel was administered at 40 mg/kg and albendazole as a single 400mg dose, in accordance with WHO guidelines [18]. Treatment exclusion criteria included children who had already received annual treatment at school, children under the age of one, seriously ill individuals and women in their first trimester of pregnancy as determined by questionnaire. For PSAC between the ages of 1 and 4 where the child was unlikely to be able to take the drug in the conventional form it was presented in a local meal called banku, made of a maize-flour paste.

### Morbidity indicators

The inclusion of the individual and household surveys allowed this study to assess for morbidity indicators in relation to WHO categorization intensities for *S*. *mansoni* and *S*. *haematobium* infections from parasitological examination [17]. Participants from the three screened communities were asked to self-report on a list of symptoms from the individual questionnaire (S2 Questionnaire, question 8).

### Statistical analysis

Descriptive statistics with 95% confidence intervals (CIs) were calculated for overall prevalence and categories for intensities as per WHO guidelines for *S*. *mansoni*, *S*. *haematobium* and STH infections. If overall prevalence was > 5%, stratified prevalences by gender, age-ranges and community were also calculated. The arithmetic mean egg count was also calculated for intensity of infection for each of the parasites, either as eggs per-gram (epg) or per 10mL.

Statistical models for prevalence of schistosomiasis in relation to WASH indicators.

To explore the relationship between the prevalence of schistosomiasis infection and WASH indicators, two different logistic generalised estimating equation (GEE) models were separately fitted for the categorical binary outcomes of *S*. *mansoni* and *S*. *haematobium* infections and WASH indicators as explanatory variables using exchangeable covariance structure. Only statistically significant covaries (i.e. by examining if p-values < = 0.0005 and their associated 95% CI) through a backward selection were retained in the final models and eventually presented. Sex and age were retained in all models. Due to the low observed STH prevalence statistical modelling was not conducted to quantify association between the prevalence of STH infection and WASH indicators.

### Multivariable Statistical models for WHO intensity categories of schistosomiasis infections in relation to morbidity indicators

To identify baseline associations between intensities of infection and morbidity indicators, two different proportional-odds cumulative logit generalized estimating equations (GEE) models were fitted to the categorical ordinal outcomes of WHO intensity categories of *S*. *mansoni* and *S*. *haematobium* infections and potential morbidity indicators as explanatory variables, using independent covariance structure. Only statistically significant covariates (i.e. by examining if p-values < = 0.005 and their associated 95% Confidence Intervals) through a backward selection were retained in the final models and eventually presented. A socioeconomic status (SES) categorical variable (for further details of how this was derived, please see relevant section below), was also tested and included as explanatory variable (or a covariate) in these models. Sex and age were always retained in the presented models.

All the GEE models (proportional odds cumulative logit and logistic ones) as described above, take into account the clustering of individuals by households. They were fitted by employing PROC GENMOD in SAS v. 9.4 (SAS Institute Inc., Cary, NC, USA) producing estimates of odds ratios (ORs) and their 95% CIs. Household-level variables were also tested and included as explanatory variables (or covariates) in these models.

### SES reduced measure

To derive an overall SES reduced measure, tested later as a covariate in the GEE proportional-odds cumulative logit models, factor analysis for categorical data of the SES questions (Q52-54, S2 Questionnaire) in the field surveys was first used. The derived factor score was then categorized based on cut-offs of tertiles of this variable into: ‘Low’, ‘Medium’ and ‘High’. Mplus software [57] was used for the factor analysis of the relevant categorical data.

## Results

### Descriptive findings

A total of 2,623 people were enrolled into the study; 878 from Tomefa, 984 from Manheam and 760 from Torgahkope-Adakope. Of those enrolled 1,834 individuals (69.9%; 95% CI = 68.1–71.7) provided stool to be screened with Kato-Katz and 1,929 (73.5%; 95% CI = 71.8–75.2) supplied urine to undergo urine filtration (Table 1).

**Table 1:** Demographics of urine and faecal samples collected by the study per site.

Site	Age Category	Urine Provided	Faeces Provided
**Tomefa**	**PSAC**	92	77
	**SAC**	233	191
	**Adults**	308	253
	**Unknown**	2	2
	**Total**	635	523
**Manheam**	**PSAC**	124	129
	**SAC**	219	205
	**Adults**	346	332
	**Unknown**	7	13
	**Total**	696	679
**Torgahkope-Adakope**	**PSAC**	110	121
	**SAC**	207	215
	**Adults**	272	286
	**Unknown**	9	10
	**Total**	598	632
**Grand Total**		1929	1834

**Fig 2:**
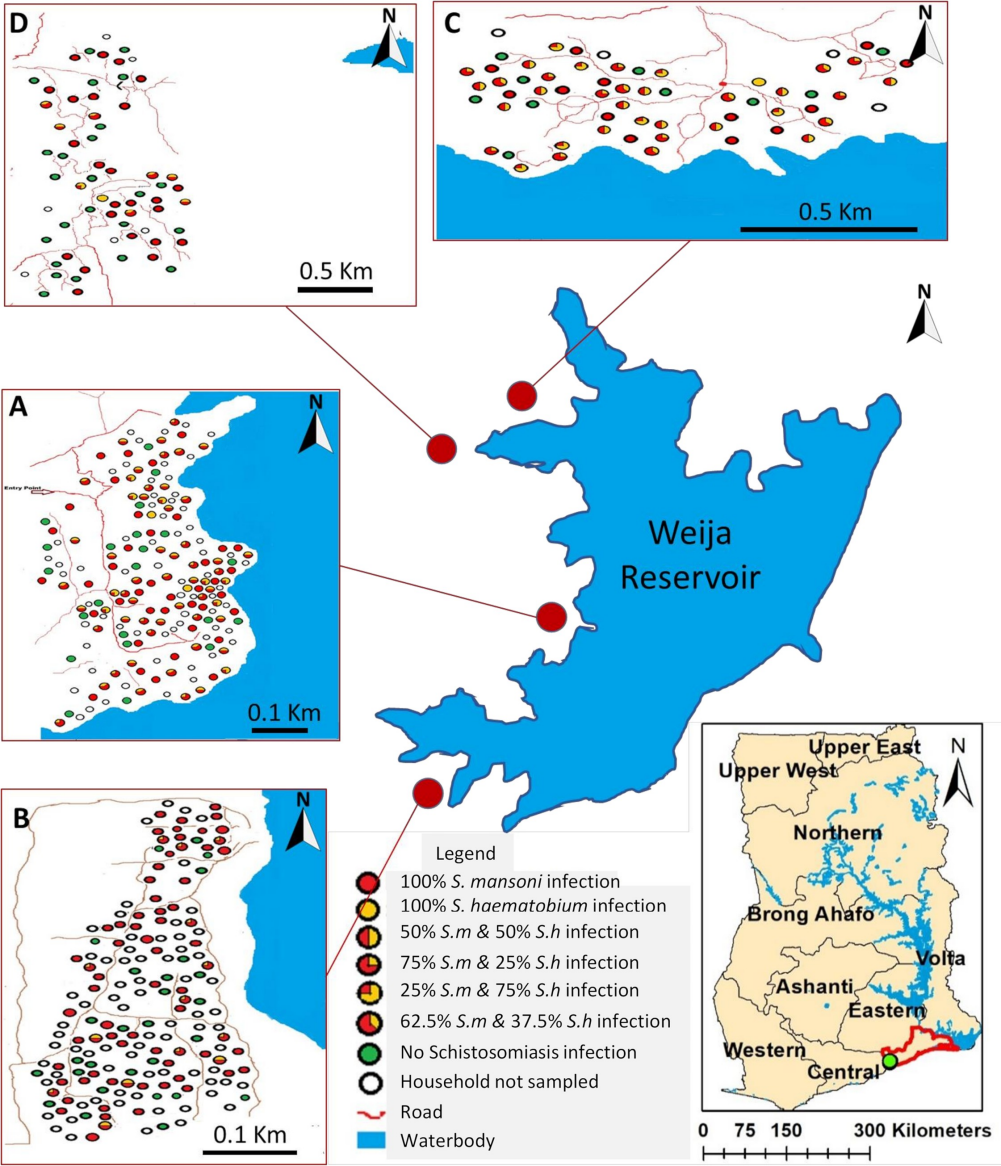
Distribution of *S*. *mansoni* and *S*. *haematobium* positive and negative households in the following sampled communities (A) Tomefa, (B) Manheam, (C) Torgahkope and (D) Adakope around the Weija reservoir, image created using ESRI ArcGIS [58].

Analysis of stool and urine samples across all three sites found the following prevalences for SCH and STH: *S*. *mansoni* 44.2% (95% CI = 41.9–46.5), *S*. *haematobium* 11.9% (95% CI = 10.4–13.3), hookworm 0.5% (95% CI = 0.2–0.9), *Trichuris trichiura* 0.4% (95% CI = 0–1.0), *Ascaris lumbricoides* 0.1% (95% CI = 0–0.3), *Strongyloides stercoralis* 0.1% (95% CI = 0–0.3) and *Taenia* s.p. 0.1% (95% CI = 0-<0.1). Whilst not listed as an STH the prevalence of *Hymenolepis* spp was observed to be 0.4% (95% CI = 0.1–0.7). The prevalence of the different helminth species across the three sampling locations is given in Table 2 and the distribution of the two schistosome species at each site is given in Fig 2.

**Table 2:** Prevalence of *Schistosoma* and STH positive samples across all three sites (CI).

	Tomefa (n = 536)		Manheam (n = 679)		TorgahKope-Adakope (n = 632)	
	n = positives	% (95% CI)	n = positives	% (95% CI)	n = positives	% (95% CI)
***S*. *mansoni***	409	78.3% (74.7–81.9)	202	30% (26.5–33.5)	196	31% (27.3–34.7)
***S*. *haematobium***	91	14.3% (11.5–17.1)	24	3.3% (1.9–4.7)	114	19% (15.8–22.2)
**Hookworm**	1	0.2% (0.0–1)	0	0	9	1.4% (0.7–2.7)
***T*. *trichiura***	0	0	3	0.4% (0.1–1.3)	0	0
***Hymenolepis spp*.**	4	0.8% (0.2–1.9)	2	0.3% (0–1.1)	0	0
***A*. *lumbricoides***	2	0.4% (0–1.4)	0	0	0	0
***S*. *stercoralis***	0	0	0	0	2	0.3% (0–1.2)
***Taenia spp*.**	0	0	1	0.1% (0–0.8)	0	0

Exploring the observed prevalence of schistosomiasis across the different age groups (PSAC, SAC and APW) within the three sampling sites, both the PSAC and APW show a high prevalence of disease. In Tomefa the prevalence of *S*. *mansoni* is significantly higher than the other two sites as is the prevalence in the adult population (Table 3).

**Table 3:** Prevalence of *S*. *haematobium* and *S*. *mansoni* infections in the three different age groups (PSAC, SAC and Adults) within each of the three sampling sites.

	Age Group	Tomefa		Manheam		TorgahKope-Adakope	
		n = positives (Total)	% (95% CI)	n = positives (Total)	% (95% CI)	n = positives (Total)	% (95% CI)
***S*. *mansoni***	Pre-School	54 (77)	70.1 (59.3–81)	9 (108)	7.9 (2.8–13.1)	13 (96)	13.7 (7.3–20.2)
	School	153 (191)	80.1 (74.5–86.3)	89 (224)	39.9 (33.1–46.8)	86 (240)	35.9 (29.2–42.5)
	Adult	200 (253)	79.1 (74.1–84.4)	107 (332)	32.1 (27–37.2)	100 (286)	34.8 (29.2–40.4)
***S*. *haematobium***	Pre-School	4 (76)	5.2 (0.2–10.2)	0 (105)	0.0 (N/A)	11 (87)	13.2 (6.5–19.8)
	School	59 (249)	23.8 (18.1–29.6)	14 (236)	5.8 (2.5–9.1)	63 (230)	27.6 (21.3–33.9)
	Adult	32 (308)	10.3 (6.7–13.8)	10 (346)	2.9 (1–4.8)	41 (272)	15.1 (10.8–19.4)

**Fig 3:**
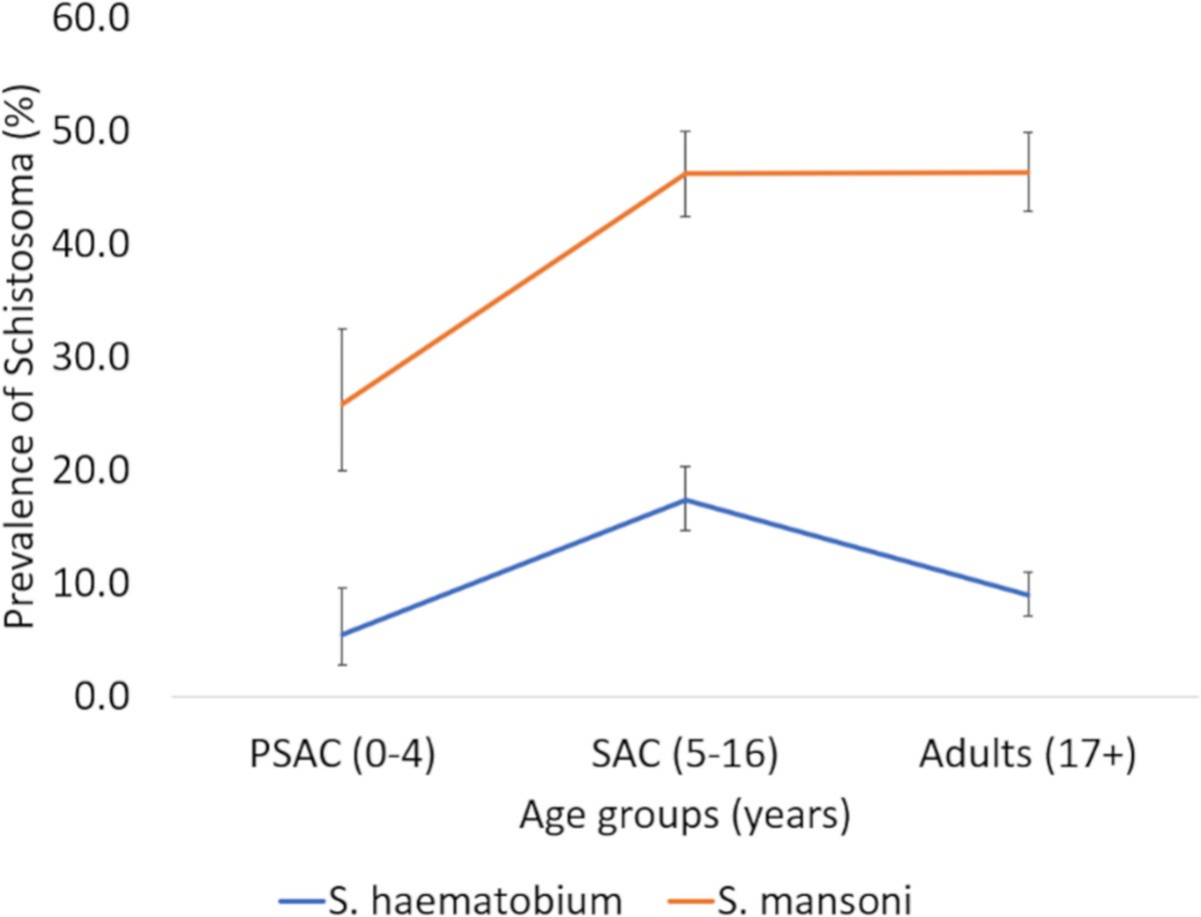
Prevalence of *Schistosoma* across different age groups for *S*. *haematobium* and *S*. *mansoni* showing the percent positive per age group.

Comparison of the percentage prevalence across different age categories reveal that both *Schistosoma* species have the highest prevalence in SAC but reduced in adulthood, however it was only in *S*. *haematobium* that this difference was significant (Fig 3).

**Fig 4:**
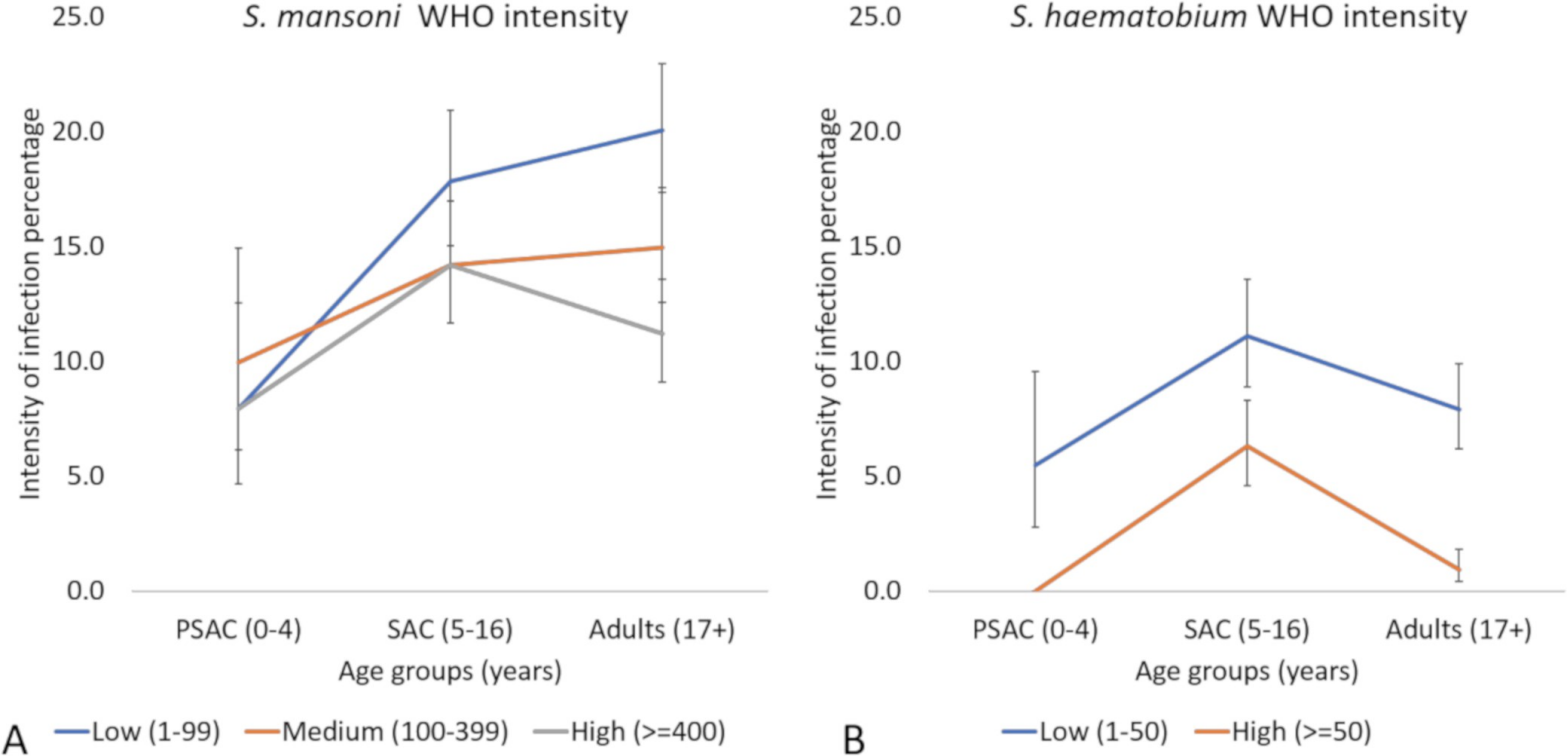
Infection intensity for (A) *S*. *mansoni* and (B) *S*. *haematobium* based on WHO egg count numbers across different age groups.

There was little difference in intensity of infection by age groups for *S*. *mansoni*. The distribution of intensity for *S*. *haematobium* infection produced a different pattern, with little difference in prevalence of low intensity infections across the three age groups. However, for high-intensity *S*. *haematobium* infections, these peaked amongst school age children and decreased in the adult population (Fig 4).

### Treatment coverage

Of the 2,623 individuals that were recruited to the study 1,840 (70.1%; 95% CI = 68.4–71.9) received PZQ treatment, of those that did not receive treatment, 99 (3.8%; 95% CI = 3–4.5) met the exclusion criteria [37], 354 (13.5%; 95% CI = 12.2–14.8) were absent, 170 refused (6.5%; 95% CI = 5.5–7.4) and for 160 (6.1%; 95% CI = 5.2–7.0) their reasons for not receiving treatment were unknown.

Similarly of the 2,623 participants enrolled in the study 1,849 (70.5%; 95% CI = 68.7–72.2) received ALB, 40 (1.5%; 95% CI = 1.1–2) met the exclusion criteria, 354 (13.5%; 95% CI = 12.2–14.8) were absent, 170 (6.5%; 95% CI = 5.5–7.4) refused and for 210 (8%; 95% CI = 7–9) their reason for not receiving treatment is unknown. The treatment of both SAC and adults in the community is given in Table 4, children treated at school, and therefore not eligible to receive further MDA in this study, were included as “treated”.

**Table 4:** Coverage of eligible PSAC, SAC and adults across all three sampling sites with regards to PZQ and ALB.

Drug	Age Group	Total	Treated	Coverage % (95% CI)
**PZQ**	**PSAC (0–4 yrs)**	278	203	73 (67.4–78.1)
	**SAC (5–17 yrs)**	519	420	80.9 (77.3–84.2)
	**Adults (18–75+ yrs)**	1474	974	66.1 (63.6–68.5)
**ALB**	**PSAC (0–4 yrs)**	403	312	77.4 (73–81.4)
	**SAC (5–17 yrs)**	565	465	82.3 (78.9–85.4)
	**Adults (18–75+ yrs)**	1284	901	70.2 (67.6–72.7)

### Models for prevalence of schistosomiasis infection and related WASH indicators

Table 5 contains results from multivariable GEE logistic models for prevalence of schistosomiasis infection related to WASH indicators. The first part of this table shows WASH associations with *S*. *mansoni* infection. Those urinating in school toilet were less likely to be infected with *S*. *mansoni* than those who did not (OR: 0.5; 95% CI = 0.3–0.8; *P* = 0.007). Furthermore, those who defecated on the ground were more likely to be infected with *S*. *mansoni* compared to those who did not (OR: 1.3; 95% CI = 1–1.6; *P* = 0.025). Those living in households with electricity were less likely to be infected with *S*. *mansoni* compared to those living in households which did not (OR: 0.2; 95% CI = 0.2–0.3, *P* = <0.001). Males were more likely to be infected with *S*. *mansoni* than females (OR: 1.8; 95% CI: 1.4–2.2; *P* = <0.001). Finally, pre-school children were less likely to be infected with *S*. *mansoni* than adults (OR: 0.3; 95% CI: 0.2–0.4; *P* = <0.001).

**Table 5:** Odds Ratios (ORs) from two separate multivariable GEE logistic models for the outcomes of *S*. *mansoni* (n = 1671) and *S*. *haematobium* (n = 1773) infections in relation to WASH indicators.

	**Comparisons between categories of covariates**	**OR, (95% CI), p-value**
*S*. *mansoni*	Sex: Males vs Females	1.8 (1.4–2.2),<0.001
	Age Category: Pre-School vs Adult	0.3 (0.2–0.4),<0.001
	Age Category: School vs Adult	1.1 (0.9–1.4),0.308
	Household electricity: Yes vs No	0.5 (0.4–0.6),<0.001
	Urinate in school toilet:Yes vs No	0.5 (0.3–0.8),0.007
	Defecate on the ground:Yes vs No	1.3 (1–1.6),0.025
	Defecate in village toilet:Yes vs No	0.5 (0.3–0.7),<0.001
	**Comparisons between categories of covariates**	**OR, (95% CI), p-value**
*S*. *haematobium*	Sex: Males vs Females	1.7 (1.5–2.3),0.001
	Age Category: Pre-School vs Adult	0.6 (0.3–1),0.041
	Age Category: School vs Adult	2.5 (1.8–3.5),<0.001
	Household electricity: Yes vs No	2.3 (1.7–3.2),<0.001
	Urinate in school toilet:Yes vs No	0.2 (0.1–0.7),0.009
	Urinate in other site:Yes vs No	2.1 (1.1–4),0.036
	Defecate in neighbor’s toilet:Yes vs No	1.9 (1.3–2.7),<0.001
	Defecate in village toilet:Yes vs No	0.1 (<0.1–0.4),<0.001

Those who urinated in school toilets and defecated in village toilets were less likely to be infected with *S*. *haematobium* than those who indicated that they did not (OR: 0.2; 95% CI: 0.1–0.7; *P* = 0.009 and OR: 0.1 95% CI: <0.1–0.5, *P* = <0.001, respectively). Those who urinated in ‘other’ sites and those who defecated in the neighbourhood toilet were more likely to be infected with *S*. *haematobium* than those who indicated that they did not (OR: 2.1; 95% CI = 1.1–4; *P* = 0.036 and OR: 1.9; 95% CI = 1.3–2.7; *P* = <0.001, respectively). Surprisingly, those living in households with electricity were more likely to be infected with *S*. *haematobium* (OR: 2.3; 95% CI = 1.7–3.2; *P* = 0.00). Males were more likely to be infected with *S*. *haematobium* than females (OR: 1.7; 95% CI = 1.3–2.3; *P* = 0.001). Compared to adults pre-school children were less likely (OR: 0.6; 95% CI = 0.3–1; *P* = 0.041) to be infected with *S*. *haematobium* while school children were more likely (OR: 2.53; 95% CI = 1.8–3.5; *P* = <0.001).

### Models for schistosomiasis related morbidity indicators

Table 6 contains results from multivariable proportional-odds cumulative logit GEE models for WHO intensity categories of *S*. *mansoni and S*. *haematobium* infections in relation to potential morbidity indicators.

**Table 6:** Odds Ratios (ORs) from two separate multivariable GEE proportional-odds cumulative logit models for the outcomes of WHO categorical intensities of *S*. *mansoni* (n = 647) and *S*. *haematobium* (n = 1586) infections in relation to morbidity indicators.

	**Comparisons between categories of covariates**	**OR, (95% CI), p-value**
*S*. *mansoni*	Blood in Stool: Currently vs Not in last month	1.7 (1–2.9),0.076
	Blood in Stool: In last month vs Not in last month	1.6 (1–2.6),0.050
	Blood in Stool: Don’t Know vs Not in last month	0.6 (0.4–1.1),0.101
	Diarrhoea: Currently vs Not in last month	1.4 (0.8–2.4),0.240
	Diarrhoea: In last month vs Not in last month	1.6 (1–2.6),0.054
	Diarrhoea: Don’t Know vs Not in last month	0.4 (0.2–0.8),0.010
	Sex: Males vs Females	2.3 (1.7–3.3),<0.001
	Age Category: Pre-School vs Adult	0.5 (0.1–2.2),0.328
	Age Category: School vs Adult	0.1 (0.0–1.1),0.056
	SES: Low vs High	2.0 (1.3–3.2),0.003
	SES: Medium vs High	1.3 (0.9–2),0.176
	Household electricity: Yes vs No	0.6 (0.4–1),0.035
	**Comparisons between categories of covariates**	**OR, (95% CI), p-value**
*S*. *haematobium*	Blood in Urine: Currently vs Not in last month	2.7 (1.6–4.6),<0.001
	Blood in Urine: In last month vs Not in last month	2.2 (1.4–3.3),0.001
	Blood in Urine: Don’t Know vs Not in last month	0.5 (0.3–0.8),0.003
	Sex: Males vs Females	1.5 (1.1–2.0),0.019
	Age Category: Pre-School vs Adult	0.7 (0.4–1.3),0.251
	Age Category: School vs Adult	2.3 (1.7–3.3),<0.001

Those replying ‘In last month’ for question ‘blood in stool’ were 1.6 times (95% CI = 1–2.6; *P* = 0.05) more likely to have higher intensities of *S*. *mansoni* infection than those replying ‘Not in last month’ for this same question. Those replying ‘In last month’ for question ‘blood in stool’ were 1.6 times (95% CI = 1–2.6; *P* = 0.05) more likely to have higher intensities of *S*. *mansoni* infection than those replying ‘Not in last month’ for this same question.

Those reporting to currently have diarrhoea or who had diarrhoea in the last month were also more likely to have a higher intensity infection of *S*. *mansoni* compared to those replying ‘Not in last month’ for this same question. Those replying ‘Currently’ and ‘In last month’ for the question ‘blood in urine’ were more likely to have higher intensities of *S*. *haematobium* infection compared to those who replied ‘Not in last month’ OR: 2.7 (95% CI = 1.6–4.6; p<0.001) and OR: 2.2 (95% CI = 1.4–3.3; p = 0.001).

Males were 2.3 times (95% CI = 1.7–3.3; *P* = <0.000) more likely to have a high intensity infection by *S*. *mansoni* and 1.5 times (95% CI = 1.1–2; *P* = 0.019) more likely to have a high intensity *S*. *haematobium* compared to females. There was no significant difference in the association of age and high intensity infection status for *S*. *mansoni* between adults and PSAC (OR: 0.5; 95% CI = 0.1–2.2; *P* = 0.33) as well as adults and SAC (OR: 0.1; 95% CI = 0–1.1; *P* = 0.06). For *S*. *haematobium* infections there was no significant difference between PSAC and adults; however, between SAC and adults, SAC were 2.3 times (*P* = <0.00; 95% CI = 1.7–3.3) more likely to be infected with a high intensity *S*. *haematobium* infection. Individuals from a low SES status compared to individuals from a high SES status were 2 times more likely being infected with *S*. *mansoni* (95% CI = 1.3–3.2; *P* = 0.00). There were not statistically significant differences in the odds of being more heavily infected with *S*. *mansoni* between individuals from medium and high SES status (OR: 1.3; 95% CI: 0.9–2; *P* = 0.18). High intensity *S*. *mansoni* infections were less likely to be found in households with electricity (OR: 0.6; 95% CI = 0.4–1; *P* = 0.04) if compared to households with no electricity.

## Discussion

### SCH and STH Prevalence

In the current study, the prevalence of schistosomiasis greatly exceeded that of STH across all three study sites, this may be due to the main economic activities in this study community being fishing as opposed to agriculture. Among the cases of schistosomiasis both urinary and intestinal schistosomiasis were observed. *S*. *mansoni* was the most prevalent across all three communities. The highest prevalence of *S*. *mansoni* was observed in Tomefa and the lowest was seen in Manheam. For *S*. *haematobium* infections, Torgahkope-Adakope had the highest prevalence with Manheam having the lowest. This difference of distribution across the sampling sites may reflect the local economic activities across the different sites, similarly the distribution of the species may reflect differences in snail habitat favouring the host of one *Schistosoma* species over another in different locations. Our study also shows that the prevalence of STH is negligible and far below the 20% recommended by WHO to justify annual MDA [16]. This low prevalence means that STH is likely to be of little health importance in the communities examined; further, there were insufficient positive samples for any statistical models to be fitted on STH outcomes in our study.

The prevalence and intensity of the disease between SAC and adults with regards to *S*. *mansoni* (Tables 5 and 6) shows no significant difference overall and at individual site level. This similarity in prevalence highlights the importance of treating the wider community, as adults in all three study villages must be contributing significantly to the local transmission of the disease. However, for PSAC with *S*. *mansoni* there are significantly more infants from Tomefa with an infection than from either Manheam or Torgahkope-Adakope, who both have similar levels of PSAC infections to each other (Table 3). This suggests that treatment strategies with regards to PSAC may need to change depending on the prevalence in communities, with PSAC at higher risk in hyperendemic areas, such as Tomefa. The same was not found to be true for *S*. *haematobium* where the prevalence was highest amongst SAC, followed by adults and then PSAC (Table 3), although it should be noted that the prevalence of *S*. *haematobium* was far lower than that of *S*. *mansoni*.

It should be noted that in calculating the prevalence and intensities, individuals that had received praziquantel in the last 12 months were included. These individuals constituted only 0.89% of recruited community members and will take into consideration this portion of our sample when we have collected follow-up data. However, we also anticipate that such low numbers will have little effect on the overall prevalence and intensities observed.

### Treatment coverage

The need to expand the treatment coverage is also important in the longer term as it increases the likelihood that elimination of these two diseases as a public health problem can be achieved in an environment where the sanitation and hygiene situation of the infected communities may not improve. Mathematical modelling studies indicate that a relatively high coverage, of 85%, amongst SAC and 40%, amongst adults, is required to bring about the 2020 goals for SCH morbidity control [23], in areas with moderate to high SCH. Despite having a high presence in the community our study fell slightly short of the coverage required for the SAC with a coverage of ~80%. The coverage of the adult population was more promising as we were able to exceed the 40%, calculated, for SCH control [23]. The largest proportion of those that did not receive treatment did so due to being absent. A possible way to increase the coverage would be to treat the participants during the survey and sample collection phase. Alternatively, leaving the drugs behind and having a community member trained in their dispensation would allow those who were absent to seek treatment if missed by the MDA. Following on from those that were absent refusal to receive treatment was the second biggest reason for people to not receive treatment. Reasons for refusal are unknown as our study did not include questions for justification of refusal. Only a small percentage were not treated due to meeting the exclusion. The number of individuals who had received treatment in the last 12 months was minimal, 0.8% (95% CI = 0.5–1.3). The results of the baseline survey reveal that the presence of STH was negligible with a combined prevalence of 1.59%, far below the 20% recommended by WHO to justify annual MDA [16].

### WASH, household, SES and morbidity indicators of schistosomiasis

Self-reported morbidity indicators were found to be significantly associated with high intensity infections (Table 6). Morbidity indicators such as blood in stool or urine and diarrhoea (also observed in our study) have been previously identified as indicators of moderate and heavy intensities of *S*. *mansoni* or *S*. *haematobium* infections [49, 59]. Being of lower socio-economic status was suggested to be a risk factor for high intensity *S*. *mansoni* infection whereas the treatment of water and the availability of household electricity were associated with a reduced intensity of infection and a lower probability of *S*. *mansoni* infection (Tables 5 and 6). Both urinating in the school toilet and defecating in the village toilet were identified as being related to lower risk of having an *S*. *haematobium* infection. Oddly household electricity was associated with an increase likelihood of being positive for *S*. *haematobium* (Table 5). The latter finding is surprising and merits further investigation to enable us to give a more reasonable explanation in the near future, but it is unlikely that having household electricity directly contributes to an increased risk of *S*. *haematobium* infection, but rather there is a secondary relationship.

Urinating and defecating in school and village toilets was associated with lower risk of schistosomiasis infection. Conversely, the use of neighbour’s toilet was linked to an increased risk of schistosomiasis infection. These patterns of toilet use are likely not directly contributing to the transmission of disease, unless these shared latrines are acting as a focal point of contamination, but instead may indicate areas with a general lack of appropriate waste disposal and sanitation infrastructure. This relates back to an increased risk of schistosomiasis as a lack of toilets could lead to behaviours such as open defecation or urination in or near waterbodies, resulting in local transmission of the disease.

Considering the high prevalence of both schistosome species, but especially *S*. *mansoni* it is surprising that no significant link was identified between schistosomiasis infection and either the nearby water reservoir, faecal/urine disposal habits or bathing habits. There was no strong link between any of the defecation or urination habits recorded by the questionnaire and an increased risk of schistosomiasis, similarly the occupation of those involved, notably fishing, was not associated with risk of infection or intensity of infection. This may be due to the answers given, the most common location for defecation, besides toilets, were the ground and the bush. These answers in no way indicate proximity to the shore of the reservoir. In total only 13 out of 2,429 individuals reported they defecated in the reservoir or a river. This discrepancy highlights the difficulty in extracting behavioural patterns from communities that sustain transmission. With the prevalence of *S*. *mansoni* as high as 78% across the entire community there must be local contamination of the immediate area with faeces. However, no behaviours associated with defecation were identified as significant risk factors for schistosomiasis infection.

### Mapping and SCH control

Our study raises questions over the suitability of mapping and surveillance methods in the context of the WHO goals for schistosomiasis control and elimination. With such a highly focal disease as schistosomiasis the ability to direct the MDA campaigns to the correct communities is crucial. This is evident in Table 2 with the different prevalences of both *S*. *mansoni* and *S*. *haematobium* differing significantly between sites. These differences in prevalence may require different intervention strategies, however at a greater scale this resolution is lost. However, being able to empirically assess each community for SCH prevalence is not possible and studies have shown that models attempting to project out the prevalence of the disease from available data are often inaccurate and operate on the wrong scale, typically at the district level [60]. The importance of precision mapping strategies are required has been identified [61].

### Study limitations

The authors accept that there are a number of limitations to their study, of importance is the use of self-reported morbidity indicators. By not having an objective morbidity assessment it is not possible to associate health problems reported by community members with schistosomiasis whilst controlling for confounders. However, the choice for self-reporting can be defended as it was pragmatic choice that has been used in other studies. Naturally with more resources it would have been possible to assess morbidity from a more objective and clinical persepective, however our study lacked the resources and time was a limiting factor, as the authors hoped to strike a balance between accuracy and reducing the time our study impacted the communities’ time.

Similarly, the socio-economic assessment could have been more detailed, which would have helped in better stratifying the communities. Only three questions were used to stratify the SES scale, inclusion of more wealth indicator may have created a higher resolution SES.

Another limitation of this study is the failure to capture reasons for treatment-refusal. Whilst the study was able to identify broad reasons for individuals not receiving treatment, (such as met exclusion criteria, was away, refused etc), the questionnaire did not capture specific reasons for refusal. Such information would have been useful in understanding how to include this group in future MDA interventions. A final limitation of the study is the potential that some communities may have been oversampled whilst others may have been under sampled, as the sample size was calculated for a community of 1,500 people in size, yet the estimated size of Torgahkope-Adakope was ~911 and that of Manheam was ~3,954. However, there is no way to know the true populations of these communities, with the estimates for population size being derived from the total number of enumerated houses multiplied by average household number per community.

### Conclusion

The three communities sampled differed in the prevalence of *S*. *mansoni* with Manheam and Torgahkope-Adakope having significantly lower prevalences than Tomefa. The prevalence of *S*. *haematobium* was significantly lower than that of *S*. *mansoni* in all three communities, although like *S*. *mansoni* the prevalence also differed by site, with Manheam having significantly fewer cases than either Torgahkope-Adakope or Tomefa. The prevalence of STH species was found to be very low in these communities, possibly due to fishing being the primary economic activity as opposed to agriculture. Individuals of a lower socioeconomic background were more likely to be infected with both species of *Schistosoma* and the prevalence of the disease was similar in both the adult and SAC populations supporting the argument that current methods of treating just the SAC members of a community are insufficient if regional elimination of SCH is to be achieved. This study also supports previous findings with regard to self-reported morbidity indicators being positively correlated with both *S*. *mansoni* and *S*. *haematobium* infections. The prevalence of schistosomiasis was also considerably high in PSAC, even to the point of no significant difference between the age groups and prevalence of *S*. *mansoni* at the Tomefa site. The issue of achieving high coverage of drug adherence is critical as it has been calculated that high coverage rates are required if the 2020 goals are to be achieved. These coverage rates were not easy to achieve amongst the SAC although the coverage rate required for adults was exceeded. Currently the major hurdles to overcome in order to expand PZQ to the whole community include data demonstrating sufficient burden to warrant treatments, scale up of the donation or acquisition of PZQ for those cohorts currently not being treated and the development of a robust method for drug distribution. A flexible enough method of drug distribution to accommodate for absenteeism, which is one of the leading causes for non-compliance, is also required. The donation and availability of a formulation of PZQ for pre-school age children is also critical, especially for the treatment of PSAC in communities with a high prevalence of disease. It is primarily these reasons that community wide MDA of PZQ is rarely implemented despite it being recommended and widely accepted by the academic community in areas of high prevalence.

Finally, the use of complimentary interventions, such as snail-control, introduction of WASH practices and improvements to infrastructure, should also be employed alongside community wide MDA, whenever possible.

## Supporting information

S1 Questionnaire The household questionnaire used to obtain data to identify risk factors associated with STH and SCH. (DOCX)

S2 Questionnaire The personal questionnaire used to obtain data to identify risk factors associated with STH and SCH. (DOCX)
