# Supplementary material for: Assessing expanded community wide treatment for schistosomiasis: Baseline infection status and self-reported risk factors in three communities from the Greater Accra region, Ghana
Source: PLoS Negl Trop Dis. 2020 Apr 27;14(4):e0007973. doi: 10.1371/journal.pntd.0007973 (PMC7205311; doi:10.1371/journal.pntd.0007973)
Supplement: S1 Questionnaire — (DOCX) [file pntd.0007973.s001.docx]

**HOUSEHOLD QUESTIONNAIRE – COMMUNITY: Schistosomiasis and soil-transmitted helminths**

**COUNTDOWN Integrated strategy for the control and elimination of schistosomiasis and soil-transmitted helminths in Ghana**

Date: (dd/mm/yy): __ __ / __ __ / __ __ **GPS coordinates** latitude: __________ longitude: __________

| *Please ask to speak to a head of the household.* Interviewer Initials | ___________________ |
| --- | --- |

| **Consent checklist:** Has written consent been obtained? Y |  | N |  | *Only proceed if Yes* |
| --- | --- | --- | --- | --- |

**______________________________________________________________________________________**

**A. Participant details**

1. Participant ID: __ __ __ / __ __ __ / __ __ 2. Village: _______________________________

| 3. Participant’s name: _____________________ | 4: Phone no: _____________________________ |
| --- | --- |

**B. Sanitation data**

| 5. Does your house have a toilet? Y |  | N |  | *If ‘N’, skip to question 11* |
| --- | --- | --- | --- | --- |

6. Can you please show me your household toilet? *Tick off (√) toilet type, using picture sheet of different toilet types as a guide:*

| Flush toilet |  |  | |  | |  | |  |  | | | |  |
| --- | --- | --- | --- | --- | --- | --- | --- | --- | --- | --- | --- | --- | --- |
| Pit latrine with cement slab/ventilated improved pit latrine |  | Flush to pit | | |  | |  | | | |  |  |  |
|  |  | Flush elsewhere | | |  | |  | | | |  |  |  |
|  |  | No water | | |  | |  | | | |  |  |  |
| Open pit/pit latrine without cement slab |  | Flush to pit | | |  | |  | | | |  |  |  |
|  |  | Flush elsewhere | | |  | |  | | | |  |  |  |
|  |  | No water | | |  | |  | | | |  |  |  |
| Other |  | Specify: |  | | | | | | |  |  |  |  |

*Tick (√) WITHOUT reading out:*

| 7. Was household toilet observed by interviewer? Y |  | N |  |
| --- | --- | --- | --- |

*Please take a photo of the household toilet*

8. *Tick off (√) all relevant observations WITHOUT reading out question:*

| Toilet is clean (no urine, faeces, flies) |  |  | | |  |
| --- | --- | --- | --- | --- | --- |
| Water or other personal cleaning materials are evident |  |  | | |  |
| There is a hole cover |  |  | | |  |
| Urine on seat |  |  | | |  |
| Faeces on seat |  |  | | |  |
| Urine on floor/walls |  |  | | |  |
| Faeces on floor/walls |  |  | | |  |
| Odour |  |  | | |  |
| Flies present |  |  | | |  |
| Other |  | Specify: |  |  |  |

| 9. Is this toilet shared with another household? | Y |  | N |  | Refused |  |
| --- | --- | --- | --- | --- | --- | --- |

*If ‘N’, skip to question 11*

10. Including yours, how many households do you share the toilet with? ____________________________

| 11. Do you have a baby or toddler in your house? Y |  | N |  | *If ‘N’, skip to question 14* |
| --- | --- | --- | --- | --- |

*(CHILDREN BETWEEN 1 AND 3 YEARS OF AGE):*

12. How do you dispose of the child’s faeces? *Tick all items mentioned or demonstrated WITHOUT reading out options:*

| In household waste |  |  | | |  |
| --- | --- | --- | --- | --- | --- |
| In the household toilet |  |  | | |  |
| In the bushes |  |  | | |  |
| In the garden |  |  | | |  |
| In the river |  |  | | |  |
| In the lake |  |  | | |  |
| Other |  | Specify: |  |  |  |
| *Don’t know* |  |  |  |  |  |
| *Refused* |  |  |  |  |  |

13. Where do you bathe your child? *Tick all items mentioned:*

| In the house |  |  | | |  |
| --- | --- | --- | --- | --- | --- |
| Using a tub in the yard |  |  | | |  |
| In the river |  |  | | |  |
| In the lake |  |  | | |  |
| Other |  | Specify: |  |  |  |

**C. Water supply data**

14. What is the main source of water for your household? *CHOOSE ONLY ONE OPTION with a tick (√)*. *Show respondent the picture sheet of different water sources. Observe the source if possible:*

| Piped water into dwelling |  |  | |  | |
| --- | --- | --- | --- | --- | --- |
| Piped water to yard/plot - this house only |  |  | |  | |
| Piped water shared with other houses |  |  | |  | |
| Piped water/tubewell/borehole or protected dug well/spring |  |  | |  | |
| Unprotected dug well/spring or surface water |  |  | |  | |
| Rainwater |  |  | |  | |
| Cart with small tank/drum or tanker-truck |  |  | |  | |
| Bottled water |  |  | |  | |
| Other |  | Specify: |  | |  |

15. Do you use the main water source (as indicated in the previous question) for: *Read all options and tick all items mentioned:*

| Drinking? |  |  | | |  |
| --- | --- | --- | --- | --- | --- |
| Cooking or Dishwashing? |  |  | | |  |
| Hand washing? |  |  | | |  |
| Bathing? |  |  | | |  |
| Laundry? |  |  | | |  |
| House cleaning? |  |  | | |  |
| Other |  | Specify: |  |  |  |

16. Where is this main water source? *CHOOSE ONLY ONE OPTION with a tick (√):*

| In your household compound |  |  | | |  |
| --- | --- | --- | --- | --- | --- |
| Elsewhere in your village |  |  | | |  |
| In a neighbouring village |  |  | | |  |
| Other |  | Specify: |  |  |  |

| 17. Approximately how far is the main water source from the house (in time)? | Less than 15 min |  |
| --- | --- | --- |
| *CHOOSE ONLY ONE OPTION with a tick (√)* | 15 min – 1 hour |  |
|  | 1 – 3 hours |  |
|  | More than 3 hours |  |

| 18. Is water always available from this main source? Y |  | N |  | *If ‘Y’, skip to question 22* |
| --- | --- | --- | --- | --- |

19. If no, how often would you say that water is not available? *CHOOSE ONLY ONE OPTION with a tick (√):*

| At least once per day |  |  | | |  |
| --- | --- | --- | --- | --- | --- |
| One or two days per week |  |  | | |  |
| More than 2 days per week |  |  | | |  |
| More than one week per month |  |  | | |  |
| More than one month per year (eg dry season) |  |  | | |  |
| Other |  | Specify: |  |  |  |

| 20. Do you store water from this main source in the household? Y |  | N |  | *If ‘N’, skip to question 22* |
| --- | --- | --- | --- | --- |

| 21. If yes, what types of container do you use to store water? *Tick all items mentioned or demonstrated:* | | | | | | | |
| --- | --- | --- | --- | --- | --- | --- | --- |
| Jerry-can |  | Covered: Y |  | N |  |  |  |
| Basin |  | Covered: Y |  | N |  |  |  |
| Other |  | Covered: Y |  | N |  | Specify: |  |

_______________________________________________________________________________________

| 22. Is there an alternative source of water that you also use for your household? Y |  | N |  |
| --- | --- | --- | --- |

*If ‘N’, skip to question 26*

23. If yes, what is this alternative source of water? *CHOOSE ONLY ONE OPTION with a tick (√).* *Show respondent the picture sheet of different water sources. Observe the source if possible:*

| Piped water into dwelling |  |  | |
| --- | --- | --- | --- |
| Piped water to yard/plot - this house only |  |  | |
| Piped water shared with other houses |  |  | |
| Tubewell or borehole or protected dug well |  |  | |
| Unprotected dug well |  |  | |
| Protected spring |  |  | |
| Unprotected spring |  |  | |
| Rainwater |  |  | |
| Cart with small tank/drum or Tanker-truer |  |  | |
| Surface water |  |  | |
| Bottled water |  |  | |
| Other |  | Specify: |  |

24. Is an alternative water source the water that you use for: *Read all options and tick (√) all items mentioned:*

| Drinking? |  |  | |  |
| --- | --- | --- | --- | --- |
| Cooking or Dishwashing? |  |  | |  |
| Hand washing? |  |  | |  |
| Bathing? |  |  | |  |
| Laundry? |  |  | |  |
| House cleaning? |  |  | |  |
| Other |  | Specify: |  | |

25. Where is this alternative water source? *CHOOSE ONLY ONE OPTION with a tick (√):*

| In your household compound |  |  | | |  |
| --- | --- | --- | --- | --- | --- |
| Elsewhere in your village |  |  | | |  |
| In a neighbouring village |  |  | | |  |
| Other |  | Specify: |  |  |  |

**_______________________________________________________________________________________**

| 26. Do you treat, boil or disinfect any of your household water? Y |  | N |  | *If ‘N’, skip to question 29* |
| --- | --- | --- | --- | --- |

27. If yes, which purposes would you treat the water for? *Read all options and tick (√) all items mentioned:*

| Drinking? |  |  | | |
| --- | --- | --- | --- | --- |
| Cooking or Dishwashing? |  |  | | |
| Hand washing? |  |  | | |
| Bathing? |  |  | | |
| Laundry? |  |  | | |
| House cleaning? |  |  | | |
| Other |  | Specify: |  |  |

28. What do you treat it with?

| Household bleach |  |  | | |  |
| --- | --- | --- | --- | --- | --- |
| Boil |  |  | | |  |
| Disinfectant |  |  | | |  |
| Filter |  |  | | |  |
| Other |  | Specify: |  |  |  |

29. Where do you tip out used water?

| On a food garden |  |  | | |  |
| --- | --- | --- | --- | --- | --- |
| On the ground next to the house |  |  | | |  |
| On the ground away from the house (at least 5 metres) |  |  | | |  |
| In the bushes |  |  | | |  |
| In a lake or stream |  |  | | |  |
| Other |  | Specify: |  |  |  |

| 30. Do you have a food garden? *(A backyard farm or small farm to grow food)* Y |  | N |  |
| --- | --- | --- | --- |

*If ‘N’, skip to question 34*

| 31. What water do you use on the garden? *Tick as appropriate:* | | |  |
| --- | --- | --- | --- |
| Rain |  |  | |
| Reused/waste water |  |  | |
| Same as main household water source |  |  | |
| Same as alternative household water source |  |  | |
| Other |  |  | |

**D. Risk factors**

| 32. Do you use human faeces in your garden as a fertilizer? Y |  | N |  |  |
| --- | --- | --- | --- | --- |

*If ‘N’, skip to question 34*

33. If yes, do you: *Tick as appropriate:*

| Put it on the garden straight away |  |  | | |  |
| --- | --- | --- | --- | --- | --- |
| Dry it out first, then put it on the garden |  |  | | |  |
| Other |  | Specify: |  |  |  |
| *Don’t know* |  |  |  |  |  |
| *Refused* |  |  |  |  |  |

34. How many animals does your household keep?

| Number of dogs |  |  | | |  |
| --- | --- | --- | --- | --- | --- |
| Number of pigs |  |  | | |  |
| Number of chickens |  |  | | |  |
| Number of cows |  |  | | |  |
| Number of goats |  |  | | |  |
| Number of horses/donkeys |  |  | | |  |
| Number of other animals |  | Specify: |  |  |  |

| 35. Where do you dispose of animal faeces? | | | *Tick as appropriate:* | | |  |
| --- | --- | --- | --- | --- | --- | --- |
| Just leave them where they are |  |  | | |  |  |
| On a food garden |  |  | | |  | |
| On a non-food garden |  |  | | |  | |
| On the ground next to the house |  |  | | |  | |
| On the ground away from the house (at least 5 metres) |  |  | | |  | |
| In the bushes |  |  | | |  | |
| In a lake or stream |  |  | | |  | |
| Other |  | Specify: | |  | |  |

_________________________________________________________________________

**E. Household characteristics**

36. What type of floor does your house have?

| Earth |  |  | | |  |
| --- | --- | --- | --- | --- | --- |
| Hardened dung |  |  | | |  |
| Cement/Concrete |  |  | | |  |
| Tile |  |  | | |  |
| Wood |  |  | | |  |
| Bricks |  |  | | |  |
| Other floor type |  | Specify: |  |  |  |

37. What type of walls does your house have?

| Wood |  |  | | |  |
| --- | --- | --- | --- | --- | --- |
| Weatherboard |  |  | | |  |
| Mud bricks |  |  | | |  |
| Mud bricks with cement |  |  | | |  |
| Oven fired bricks |  |  | | |  |
| Cement blocks |  |  | | |  |
| Stones |  |  | | |  |
| Bamboo |  |  | | |  |
| Tin sheets |  |  | | |  |
| Palm leaf/raffia |  |  | | |  |
| Other |  | Specify: |  |  |  |

38. How many rooms does your household have? ____________________________

40. Does your household own any of the following types of transport? *Read all options and tick (√) all items mentioned:*

| Bicycle | Y |  | N |  |  |
| --- | --- | --- | --- | --- | --- |
| Motorbike | Y |  | N |  |  |
| Animal drawn cart | Y |  | N |  |  |
| Car | Y |  | N |  |  |
| Truck | Y |  | N |  |  |
| Fishing boat | Y |  | N |  |  |
| Engine boat (outboard motor) | Y |  | N |  |  |
| Other | Y |  | Specify: | |  |

| 43. Do you have electricity/solar panels at home? Y |  | N |  |
| --- | --- | --- | --- |

44. Does your household have any of the following home appliances that work? *Tick as appropriate:*

| Gas stove | Y |  | N |  |
| --- | --- | --- | --- | --- |
| Electrical lights | Y |  | N |  |
| Electric stove | Y |  | N |  |
| Television | Y |  | N |  |
| Refrigerator | Y |  | N |  |
| Radio set | Y |  | N |  |
| Washing machine | Y |  | N |  |
| Internet service | Y |  | N |  |
| Computer | Y |  | N |  |

45. How many mobile phones do you have? ____________________________

This completes the questionnaire. We are grateful for your participation - thank you.
