# Supplementary material for: Assessing expanded community wide treatment for schistosomiasis: Baseline infection status and self-reported risk factors in three communities from the Greater Accra region, Ghana
Source: PLoS Negl Trop Dis. 2020 Apr 27;14(4):e0007973. doi: 10.1371/journal.pntd.0007973 (PMC7205311; doi:10.1371/journal.pntd.0007973)
Supplement: S2 Questionnaire — (DOCX) [file pntd.0007973.s002.docx]

**INDIVIDUAL QUESTIONNAIRE – COMMUNITY: Schistosomiasis and soil-transmitted helminths**

**COUNTDOWN Integrated strategy for the control and elimination of schistosomiasis and soil-transmitted helminths in Ghana**

Date: (dd/mm/yy): __ __ / __ __ / __ __ Interviewer Initials ___________

**Consent checklist**

| Has written consent been obtained? Y |  | N |  | *Only proceed if Yes* |
| --- | --- | --- | --- | --- |

**____________________________________________________________________________________**

**A. Participant details**

1. Participant ID: __ __ __ / __ __ __ / __ __ 2. Village ____________________________________

| 3. Participant’s name: _________________________ | 4: Gender: Male |  | Female |  |  |
| --- | --- | --- | --- | --- | --- |

| 5. Is full date of birth known? Y |  | N |  |
| --- | --- | --- | --- |

| 6. Date of birth (dd/mm/yy): __ __ / __ __ / __ __ | 7: Current age: ________ |
| --- | --- |

**ALL INDIVIDUALS AGED 12 YEARS AND OVER ARE ABLE TO COMPLETE THIS QUESTIONNAIRE FOR THEMSELVES**

**IF THEY WISH TO, OTHERWISE THE PARENT/CAREGIVER CAN DO IT. WHERE A PARENT IS COMPLETING A QUESTIONNAIRE FOR THEIR CHILD, QUESTIONS 4 AND 5 MUST BE COMPLETED.**

8. Parent/guardian participant ID: __ __ __ / __ __ __ / __ __

9. Relationship with participant:

| Mother |  |  | | |  |
| --- | --- | --- | --- | --- | --- |
| Father |  |  | | |  |
| Aunt |  |  | | |  |
| Uncle |  |  | | |  |
| Older sister |  |  | | |  |
| Older brother |  |  | | |  |
| Grandmother |  |  | | |  |
| Grandfather |  |  | | |  |
| Other relative |  | Specify: |  |  |  |

**B. Antiparasitic drugs**

| 10. To your knowledge, have you taken albendazole (deworming tablet) in the last 12 months? Y | | | | | | | | | | | | | |  | | N | | | |  | |  |
| --- | --- | --- | --- | --- | --- | --- | --- | --- | --- | --- | --- | --- | --- | --- | --- | --- | --- | --- | --- | --- | --- | --- |
| 11. To your knowledge, have you taken praziquantel (deworming tablet) in the last 12 months? Y | | | | | | | | | | | | | | |  | | | N | | |  | |
| 12. To your knowledge, have you taken antimalarial medication in the last 12 months? Y | | | | | | | | | |  | | N | | | |  | | |  |  |  |  |
| 13. To your knowledge, have you had malaria, or a test for malaria, in the last 2 months? Y | | | | | | | | | | |  | | N | | | |  | |  |  |  |  |
| 14. Did you sleep under a bed-net last night? Y |  | N |  | |  |  |  |  |  |  |  |  |  |  |  |  |  |  |  |  |  |  |
| 15. Do you have any access to treatments for deworming? Y | | | |  | | N |  |  |  |  |  |  |  |  |  |  |  |  |  |  |  |  |

*If ‘N’, skip to question 17*

16. Where do you get deworming treatments? *Tick all items mentioned:*

| My village |  |  | | |  |
| --- | --- | --- | --- | --- | --- |
| Other village |  | Village name: |  |  |  |
| School |  | School name: |  |  |  |
| Aid post |  |  |  |  |  |
| Health centre |  |  |  |  |  |
| Pharmacy |  |  |  |  |  |
| Shop |  |  |  |  |  |
| Other deworming place |  | Specify: |  |  |  |

**C. General health data**

17-43. For the following symptoms, please indicate whether you are experiencing them currently, or have experienced them in the last month? *Tick (√) all items mentioned or demonstrated:*

|  | Symptom | Currently | In last month | Not in last month |
| --- | --- | --- | --- | --- |
| 17. | Diarrhoea (>3 watery stools a day – takes the form of the container) |  |  |  |
| 18. | Abdominal distension/swelling |  |  |  |
| 19. | Abdominal cramping/pain |  |  |  |
| 20. | Vomiting |  |  |  |
| 21. | Blood in stool |  |  |  |
| 22. | Blood in urine (not menstrual for women) |  |  |  |
| 23. | Painful urination |  |  |  |
| 24. | More frequent or urgent urination than usual |  |  |  |
| 25. | Pelvic pain |  |  |  |
| 26. | Lower back pain |  |  |  |
| 27. | Fever/headache |  |  |  |
| 28. | Malaise/weakness |  |  |  |
| 29. | Body aches/joint pains |  |  |  |
| 30. | Itchy rash |  |  |  |
| 31. | Other (please specify): |  |  |  |

**D. Personal hygiene data**

32. What is your main source of drinking water? *CHOOSE ONLY ONE OPTION with a tick (√)*. *Show respondent the picture sheet of different water sources. OBSERVE THE SOURCE if possible:*

| Piped water into dwelling |  |  | | |  |
| --- | --- | --- | --- | --- | --- |
| Piped water to yard/plot - this house only |  |  | | |  |
| Piped water shared with other houses |  |  | | |  |
| Tubewell or borehole or protected dug well |  |  | | |  |
| Unprotected dug well |  |  | | |  |
| Protected spring |  |  | | |  |
| Unprotected spring |  |  | | |  |
| Rainwater |  |  | | |  |
| Cart with small tank/drum or tanker-truck |  |  | | |  |
| Surface water |  |  | | |  |
| Bottled water |  |  | | |  |
| Other water source |  | Specify: |  |  |  |

*Tick (√) WITHOUT reading out:*

| 33. Was water source observed by interviewer? Y |  | N |  |
| --- | --- | --- | --- |

| 34. Do you swim or play in a lake or river? | Y |  | N |  | If yes, where? |  |
| --- | --- | --- | --- | --- | --- | --- |

*If ‘N’, skip to question 36*

35. If yes, how often do you swim or play there?

| Every day in the morning |  |  | | |  |
| --- | --- | --- | --- | --- | --- |
| Every day in the afternoon |  |  | | |  |
| Every day morning and afternoon |  |  | | |  |
| 1-2 times per week |  |  | | |  |
| 1-2 times per fortnight |  |  | | |  |
| 1-2 times per month |  |  | | |  |
| Other time |  | Specify: |  |  |  |

36. Would you explain and show me what you use when you wash your hands? *Tick (√) all items mentioned or demonstrated WITHOUT reading out options:*

| Uses soap/ash |  |  | | |  |
| --- | --- | --- | --- | --- | --- |
| Does not use soap/ash |  |  | | |  |
| Other |  | Specify: |  |  |  |

37. Would you explain and show me what you do when you dry your hands after washing? *Tick (√) all items mentioned or demonstrated WITHOUT reading out options:*

| Dries hands hygienically – by using a clean cloth |  |  | | |  |
| --- | --- | --- | --- | --- | --- |
| Dries in clothes |  |  | | |  |
| Does not dry hands (e.g. air drying) |  |  | | |  |
| Other |  | Specify: |  |  |  |

38. When do you wash your hands? *Tick (√) all items mentioned WITHOUT reading out options:*

| After defecation or urination |  |  | | |  |
| --- | --- | --- | --- | --- | --- |
| After cleaning babies’ bottoms/ disposal of children’s faeces |  |  | | |  |
| Before food preparation |  |  | | |  |
| Before eating/ Before feeding children |  |  | | |  |
| After contact with animals |  |  | | |  |
| After contact with soil or dirt |  |  | | |  |
| Other |  | Specify: |  |  |  |

**E. Sanitation data**

39-41. Do you wear shoes for the following? *CHOOSE ONLY ONE OPTION FOR EACH:*

|  |  | Always | Sometimes | Never |
| --- | --- | --- | --- | --- |
| 39. | When inside your home |  |  |  |
| 40. | When outside your home |  |  |  |
| 41. | When defecating/urinating |  |  |  |

42. Where is the main place you go to urinate? *Tick (√) WITHOUT reading out options:*

| Household toilet |  |  | | |  |
| --- | --- | --- | --- | --- | --- |
| Village toilet |  |  | | |  |
| School toilet |  |  | | |  |
| Neighbour toilet |  |  | | |  |
| On the ground |  |  | | |  |
| In the river |  |  | | |  |
| In the lake |  |  | | |  |
| Other site |  | Specify: |  |  |  |
| *Don’t know* |  |  |  |  |  |
| *Refused* |  |  |  |  |  |

43. Where is the main place you go to defecate? *Tick (√) WITHOUT reading out options:*

| Household toilet |  |  | | |  |
| --- | --- | --- | --- | --- | --- |
| Village toilet |  |  | | |  |
| School toilet |  |  | | |  |
| Neighbour toilet |  |  | | |  |
| On the ground |  |  | | |  |
| In the river |  |  | | |  |
| In the lake |  |  | | |  |
| Other site |  | Specify: |  |  |  |
| *Don’t know* |  |  |  |  |  |
| *Refused* |  |  |  |  |  |

| 44. Does your house have a toilet? | Y |  | N |  | 45. If yes, do you use it? | Y |  | N |  |
| --- | --- | --- | --- | --- | --- | --- | --- | --- | --- |

*If ‘Y’, skip to question 47*

46. If no, why don’t you use it? *Tick all items mentioned or demonstrated WITHOUT reading out options:*

| Females not permitted when menstruating OR not suitable for both genders |  |  | | |  |
| --- | --- | --- | --- | --- | --- |
| Toilet is dirty |  |  | | |  |
| Toilet is broken |  |  | | |  |
| Toilet pit is full/overflowing |  |  | | |  |
| Toilet is not suitable for children |  |  | | |  |
| Toilet is in an unsafe location |  |  | | |  |
| Must pay a fee to use it |  |  | | |  |
| Toilet is too far away |  |  | | |  |
| Other |  | Specify: |  |  |  |
| *Don’t know* |  |  |  |  |  |
| *Refused* |  |  |  |  |  |

47. [Besides your household toilet,] where else do you usually defecate/urinate? *Tick (√) WITHOUT reading out options:*

| Nowhere else |  |  | | |  |
| --- | --- | --- | --- | --- | --- |
| Village toilet |  |  | | |  |
| School toilet |  |  | | |  |
| Neighbour toilet |  |  | | |  |
| On the ground |  |  | | |  |
| In the river |  |  | | |  |
| In the lake |  |  | | |  |
| Other site |  | Specify: |  |  |  |
| *Don’t know* |  |  |  |  |  |
| *Refused* |  |  |  |  |  |

48. What do you clean yourself with after defecating? *Tick all items mentioned or demonstrated WITHOUT reading out options:*

| Use toilet paper |  |  | | |  |
| --- | --- | --- | --- | --- | --- |
| Use newspaper |  |  | | |  |
| Use leaves or sticks |  |  | | |  |
| Use water and hand |  |  | | |  |
| Use corn cobs |  |  | | |  |
| Use stones |  |  | | |  |
| Other cleansing type |  | Specify: |  |  |  |
| *Don’t know* |  |  |  |  |  |
| *Refused* |  |  |  |  |  |

| 49. Does your village have a public toilet? | Y |  | N |  | 50. If yes, do you use it? | Y |  | N |  |
| --- | --- | --- | --- | --- | --- | --- | --- | --- | --- |

51. If no, why don’t you use it? *Tick all items mentioned or demonstrated WITHOUT reading out options:*

| Females not permitted when menstruating OR not suitable for both genders |  |  | |  | |
| --- | --- | --- | --- | --- | --- |
| Toilet is dirty |  |  | |  | |
| Toilet is broken |  |  | |  | |
| Toilet pit is full/overflowing |  |  | |  | |
| Toilet is not suitable for children |  |  | |  | |
| Toilet is in an unsafe location |  |  | |  | |
| Must pay a fee to use it |  |  | |  | |
| Toilet is too far away |  |  | |  | |
| Other |  | Specify: |  | |  |

**F. Socio-economic characteristics –** *Complete* *ONLY IF* *18 years and over*

52. What is your highest level of education? (tick only one option)

| Never went to school |  |
| --- | --- |
| Preschool |  |
| Completed primary school (6 years) |  |
| Completed secondary/high (12 years) |  |
| Completed professional training |  |
| Completed university |  |
| Other |  |
| *Don’t know* |  |
| *Refused* |  |

53. What is your current employment status?

| Employed/has job |  | Specify job: | Farming/agriculture |  |  |  |
| --- | --- | --- | --- | --- | --- | --- |
| Doing housework |  |  | Fishing |  |  |  |
| Student |  |  | Animal Keeper |  |  |  |
| Retired |  |  | Clerk/administration |  |  |  |
| Long-term disabled |  |  | Health worker |  |  |  |
| Unemployed |  |  | Selling at market |  |  |  |
| *Don’t know* |  |  |  |  |  |  |
| *Refused* |  |  | Other |  | Specify: |  |
|  |  |  | *Refused* |  |  | |

54. How much income did your household receive over the last year?

| Less than USD $365 |  |
| --- | --- |
| USD $365-730 |  |
| USD $730-1460 |  |
| More than USD $1460 |  |
| *Don’t know* |  |
| *Refused* |  |

_______________________________________________________________________________________

**G. School characteristics –** *Complete* *ONLY IF* *aged 5-17 years…*

| 55. Do you go to school? Y |  | N |  | *If ‘N” skip to end.* |
| --- | --- | --- | --- | --- |

56. Which school do you go to: _____________________________________________

57-59. Do you wear shoes for the following? *CHOOSE ONLY ONE OPTION FOR EACH:*

|  |  | Always | Sometimes | Never |
| --- | --- | --- | --- | --- |
| 57. | When inside your classroom |  |  |  |
| 58. | When outside your classroom |  |  |  |
| 59. | When defecating/urinating at school |  |  |  |

| 60. Does your school have a toilet? Y |  | N |  | *If ‘N” skip to end.* |
| --- | --- | --- | --- | --- |

61. If yes, what type of toilet? *Show respondent the picture sheet of different toilet types. Observe school toilet if possible.*

| Flush toilet |  |  | | | | | |  |  | |  | | | |  | | | | |  | |
| --- | --- | --- | --- | --- | --- | --- | --- | --- | --- | --- | --- | --- | --- | --- | --- | --- | --- | --- | --- | --- | --- |
| Pit latrine |  | With slab | | |  | Flush to pit | | | |  | |  | | | |  | | | | |  |
|  |  | Without slab | | |  | Flush elsewhere | | | |  | |  | | | |  | | | | |  |
|  |  |  |  | | | | |  | No water |  | |  | | | | | |  | | |  |
| Composting toilet |  |  | |  | | |  | | | | | |  |  | | |  | |  |  |  |
| Bucket |  |  | |  | | |  | | | | | |  |  | | |  | |  |  |  |
| Hanging toilet or latrine |  |  | |  | | |  | | | | | |  |  | | |  | |  |  |  |
| Other |  | Specify: | |  | | | | | | | | | | | | | | |  |  |  |

*Tick (√) WITHOUT reading out:*

| 62. Was school toilet observed by interviewer? Y |  | N |  |
| --- | --- | --- | --- |

| 63. Do you use the school toilet? | Y |  |  | N |  | Refused |  |  | *If ‘Y’, skip to end* |
| --- | --- | --- | --- | --- | --- | --- | --- | --- | --- |

64. If no, why is this? *Tick all items mentioned*

| Girls are not permitted to use it OR it is not suitable for both genders |  |  | | |  |
| --- | --- | --- | --- | --- | --- |
| Toilet is dirty |  |  | | |  |
| Toilet is broken |  |  | | |  |
| Toilet pit is full/overflowing |  |  | | |  |
| Toilet is not safe for children |  |  | | |  |
| Other |  | Specify: |  |  |  |
| *Don’t know* |  |  |  |  |  |
| *Refused* |  |  |  |  |  |

_______________________________________________________________________________________

This completes the questionnaire. We are grateful for your participation - thank you.
